# Multimodal MRI Analysis of Cervical Cancer on the Basis of Artificial Intelligence Algorithm

**DOI:** 10.1155/2021/1673490

**Published:** 2021-11-08

**Authors:** Bin Wang, Yuanyuan Zhang, Chunyan Wu, Fen Wang

**Affiliations:** ^1^Department of Obstetrics and Gynecology, Xi'an Daxing Hospital, Xi'an 710000, Shaanxi, China; ^2^Department of Obstetrics and Gynecology, Affiliated Hospital of Yan'an University, Yan'an 716000, Shaanxi, China; ^3^Department of Gynaecology, Yan'an Hospital of Traditional Chinese Medicine, Yan'an 716000, Shaanxi, China

## Abstract

The purpose of this study is to explore the application value of artificial intelligence algorithm in multimodal MRI image diagnosis of cervical cancer. Based on the traditional convolutional neural network (CNN), an artificial intelligence 3D-CNN algorithm is designed according to the characteristics of cervical cancer. 70 patients with cervical cancer were selected as the experimental group, and 10 healthy people were selected as the reference group. The 3D-CNN algorithm was applied to the diagnosis of clinical cervical cancer multimodal MRI images. The value of the algorithm was comprehensively evaluated by the image quality and diagnostic accuracy. The results showed that compared with the traditional CNN algorithm, the convergence rate of the loss curve of the artificial intelligence 3D-CNN algorithm was accelerated, and the segmentation accuracy of whole-area tumors (WT), core tumor areas (CT), and enhanced tumor areas (ET) was significantly improved. In addition, the clarity of the multimodal MRI image and the recognition performance of the lesion were significantly improved. Under the artificial intelligence 3D-CNN algorithm, the Dice values of WT, ET, and CT regions were 0.78, 0.71, and 0.64, respectively. The sensitivity values were 0.92, 0.91, and 0.88, respectively. The specificity values were 0.93, 0.92, and 0.9 l, respectively. The Hausdorff (Haus) distances were 0.93, 0.92, and 0.90, respectively. The data of various indicators were significantly better than those of the traditional CNN algorithm (*P* *<* 0.05). In addition, the diagnostic accuracy of the artificial intelligence 3D-CNN algorithm was 93.11 ± 4.65%, which was also significantly higher than that of the traditional CNN algorithm (82.45 ± 7.54%) (*P* *<* 0.05). In summary, the recognition and segmentation ability of multimodal MRI images based on artificial intelligence 3D-CNN algorithm for cervical cancer lesions were significantly improved, which can significantly enhance the clinical diagnosis rate of cervical cancer.

## 1. Introduction

Cervical cancer is one of the most common gynecologic malignancies worldwide, with high morbidity and mortality, and there is a huge population of patients in China [1]. Currently known cervical cancer treatment factors include human papillomavirus (HPV) infection, chlamydia infection, smoking, overweight or unhealthy lifestyle, and the use of intrauterine devices [2–4]. Timely regular screening and early diagnosis are important for the prevention and treatment of cervical cancer because precancerous lesions can occur before cervical cancer occurs and can develop into cancer for years [5, 6]. Among the existing imaging diagnostic techniques, ultrasound is widely used in the screening of cervical cancer because of its convenience and low cost. CT has a high-density resolution and can clearly show organs and soft tissue structures with small density differences. However, it cannot judge the infiltration and metastasis of cervical cancer in other locations, so its clinical application is limited [7]. Due to the advantages of multiparameter multisequence imaging and high tissue resolution, MRI plain scan is very suitable for the diagnosis and staging of cervical cancer [8], but there are still limitations. In recent years, a variety of new multimodal MRI sequences emerged, which greatly improved the diagnostic accuracy of MRI images of various diseases.

However, the traditional artificial multimodal MRI image diagnosis often requires professional doctors to manually outline the lesion area according to the effective information in the image, and there is a certain degree of image recognition and doctor experience difference, which makes the medical image segmentation technology based on the computer-aided artificial intelligence algorithm become one of the key directions of development [9]. Among the reported methods for regional segmentation of medical images based on artificial intelligence, convolutional neural network (CNN) algorithms based on deep learning emerge endlessly [10, 11], and artificial intelligence learning algorithms such as clustering and random forest classifier are often used to solve the problem of tumor segmentation [12, 13]. However, there is no report on the related research of artificial intelligence algorithm for multimodal MRI images of cervical cancer. Therefore, this study hopes to design an artificial intelligence 3D-CNN algorithm for the lesion image characteristics of cervical cancer patients under multimodal MRI and apply it to the diagnosis of multimodal MRI images of clinical cervical cancer. The application potential of the algorithm is comprehensively evaluated by evaluating the performance of the algorithm and comparing the diagnostic accuracy.

## 2. Materials and Methods

### 2.1. Research Objects

Seventy patients with cervical cancer diagnosed in hospital from March 2018 to March 2021 were selected as the subjects. All patients underwent an MRI examination. The age of patients ranged from 34 to 75 years. The average age was (48.63 ± 6.35) years old. The clinical stages of all patients were as follows: 18 patients in stage 0, 5 patients in stage IA, 12 patients in stage IB, 11 patients in stage II A, 7 patients in stage II B, 3 patients in stage III, and 12 patients in stage IV. There was no significant difference in general clinical data such as age, height, weight, and combined diseases among all patients.

The inclusion criteria of patients were as follows: (1) patients were not treated with radiotherapy or chemotherapy or surgery before MRI examination; (2) patients had complete medical records; and (3) patients signed informed consent. Exclusion criteria were as follows: (1) image blur did not meet the requirements and patients with cervical polyps, cysts; (2) the image quality was poor and cannot meet the minimum technical requirements; and (3) patients with incomplete medical records or without signing informed consent.

All procedures of this study were approved by the ethics committee of the hospital, and the subjects included in the study signed informed consent.

### 2.2. MRI Scanning Equipment and Parameters

All patients in this study (set as the experimental group) needed to fast for 6 h before MRI scanning, relieve the bowel, fill the bladder appropriately, and receive a certain degree of respiratory training before examination. In addition, the parameters of multimodal MRI scanning in 10 normal subjects (set as the reference group) were taken as the reference, and the parameters of multimodal MRI scanning as the control of this experiment were obtained.

The magnetic resonance scanning equipment used in this study was Siemens 3.0 T magnetic resonance scanner, Germany, and 18-channel surface phased array coils were used to cover the pelvic cavity of patients. All patients underwent conventional MRI plain scan and functional MRI scan. Conventional MRI sequences included sagittal, coronal, and axial T2W1 sequences and axial T1WI sequences. Functional MRI sequences included DW1 sequence, IVIM sequence, and DCE-MRI sequence. MRI plain scan sequence scanning parameters included layer spacing of 0.8 mm, layer thickness of 4.0 mm, TE 85 ms, TR 3500 ms, and FOV 300 mm × 300 mm. IVIM sequence scan parameters included layer spacing of 1 mm, layer thickness of 5.0 mm, TE 60 ms, TR 4800 ms, and FOV 380 mm × 380 mm. DCE-MRI sequence: T1WI-TWIST: inversion angles of 20 and 150, layer spacing of 0.7 mm, layer thickness of 3.5 mm, TE 1.90 ms, TR 4.91 ms, and FOV 260 mm × 260 mm. A high pressure intravenous contrast agent was used, with an injection volume of 0.2 mL/kg at a flow rate of 3 mL/s. 20 mL of saline was injected into the tube. After 5 stages, gadolinium DTPA was injected, and the same amount of 20 mL of saline was injected into the tube. A total of 70 periods are required, with time resolution 5s and scanning time 375s.

### 2.3. Establishment of Cervical Cancer Lesion Segmentation Model Based on Artificial Intelligence 3D-CNN Algorithm

In this study, based on the traditional CNN, the time dimension is introduced into the convolution kernel operation. On the basis of retaining the characteristic information of the input data, three-dimensional data are added, so that the output data after sliding window operation are still arranged in three-dimensional space.

The basic framework of the algorithm is constructed according to the CNN structure. The feature extractor is deployed in the overall structure of the network to make the input data enter the network. After the convolution layer, pooling layer, and nonlinear function activation layer, the characteristics of different levels of the image are gradually extracted. The calculation of convolution layer is

(1) $$\begin{array}{c} K=k\left(K_{a-1}\right)*D_{a}+B_{a}, \end{array}$$

where *K*_*a*−1_ represents the information of the feature map of the upper layer, *B*_*a*_ represents the offset, *D*_*a*_ represents the weight matrix, and each row in the matrix corresponds to the weight of neurons connected to all neurons of the upper layer. The nonlinear activation function, ReLU function, is introduced between the upper and lower input in the activation layer, and its mathematical expression is shown as follows:

(2) ReLU=max0,x.

Since the multimodal MRI image segmentation task of cervical cancer patients in this study has four output categories, to solve the problem of uneven categories in the data processing process, this study introduces the Dice loss function to be applied to the cervical cancer tumor image segmentation task. The Dice score coefficient (DSC) used is an evaluation index based on the pixel overlap mentioned above. The calculation method is shown as follows:

(3) $$\begin{array}{c} D=\frac{2\sum_{j}^{M}g_{j}h_{j}}{\sum_{j}^{M}g_{j}^{2}+\sum_{j}^{M}h_{j}^{2}}, \end{array}$$

where *g*_*j*_ represents the pixel value of point *j* in the prediction results and *h*_*j*_ represents the pixel value of point *j* in the truth label and calculates the sum of *M* pixels. The gradient calculation is shown as follows:

(4) $$\begin{array}{c} \frac{\partial D}{\partial g_{i}}=2\left[\frac{h_{i}\left(\sum_{j}^{M}g_{j}^{2}+\sum_{j}^{M}h_{j}^{2}\right)-2g_{i}\sum_{j}^{M}g_{j}h_{j}}{\sum_{j}^{M}g_{j}^{2}+\sum_{j}^{M}h_{j}^{2}}\right], \end{array}$$

where *i* represents the *i* pixel. Accordingly, the loss function for cervical cancer image segmentation can be expressed as

(5) $$\begin{array}{c} N=\frac{2}{|F|}\sum f\in F\frac{\sum_{j}g_{j,f}h_{j,f}}{\sum_{j}g_{j}+\sum_{j}h_{j}}, \end{array}$$

where *F* represents the category; *f* represents a category in the cervical cancer tumor segmentation task in this study; *g* represents the segmentation result region predicted by the network; *h* represents the segmentation region calibrated by the true value label; *j* represents any pixel in the image; and *g*_*i*,*f*_ and *h*_*i*, *f*_ represent the numerical parameters of the predicted output and the pixel output in the true value label region at *j*.

In order to adapt to the dense connection operation in 3D-CNN, the jump structure is used to connect the feature maps of each layer in this study [14], and then the feature maps of all layers are connected in series. The corresponding expression is shown as follows:

(6) $$\begin{array}{c} S_{1}=H\left(S_{0},S_{1},S_{2},\ldots ,S_{j-1}\right). \end{array}$$

Then, the residual structure is introduced to solve the gradient dispersion problem in the data processing of deep CNN. In essence, the residual structure adds a bypass to make a sum operation between the input data and the output data and then constructs a deep network model, which is also called shortcut structure. The output expression of the residual unit is shown as follows:

(7) $$\begin{array}{c} Y_{1}=H\left(S_{0}\right)+F\left(S_{1},\omega_{1}\right), \end{array}$$

where *H*(·) represents the fitting target in the CNN, *F*(*S*_1_, *ω*_1_) represents the convolution operation, *ω*_1_ represents the weight parameter in the network, and *Y*_1_ represents the network output of the first layer. If *H*(·) is an identity mapping, which is expressed as (8), the network output *Y*_1_ of the residual structure of the first layer can be expressed as equation (9):

(8) $$\begin{array}{c} H\left(S_{1}\right)=S_{1}, \end{array}$$

(9) $$\begin{array}{c} Y_{1}=S_{1}+1=S_{1}+F\left(S_{1},\omega_{1}\right). \end{array}$$

The overall output of the residual structure unit is a linear sum of the original data of the input image and the output data after the convolution operation. In this way, the output of the entire network can be represented by a sum algorithm. It is assumed that there are a total of *M* residual structure network units in the network, and the overall output of the residual network can be expressed as follows:

(10) $$\begin{array}{c} S_{M}=S_{m}+\overset{M-1}{\underset{j=m}{\sum }}F\left(S_{1},\omega_{1}\right). \end{array}$$

**Figure 1:**
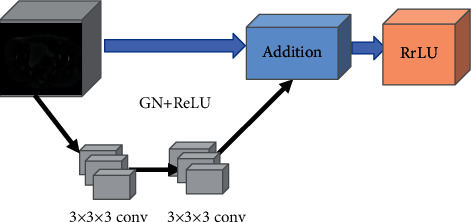
Residual unit structure.

The convolution layer in the residual structure unit used in this study is set to a three-dimensional convolution mode, and the BN (Batch Normalization) layer is changed to the GN (Group Normalization) layer operation (Figure 1).

**Figure 2:**
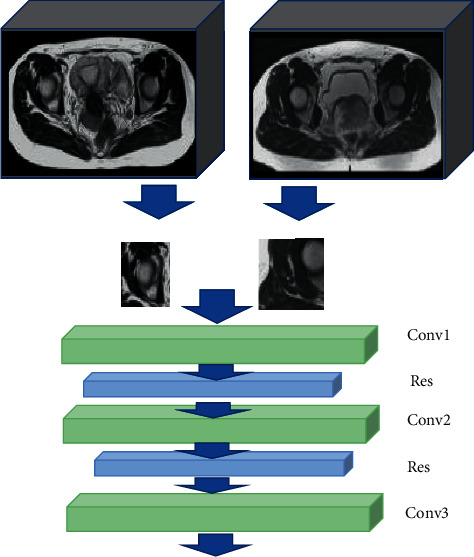
Network structure pattern of cervical cancer characteristic lesions extraction.

**Figure 3:**
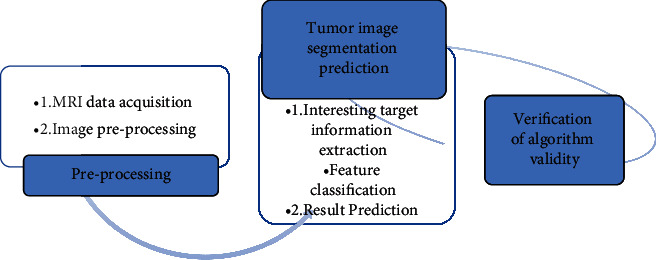
Flowchart based on artificial intelligence 3D-CNN algorithm.

The convolution layer with the size of the convolution kernel is set to 3 × 3 × 3, and then the GN layer is used to accelerate the convergence of the network. Then, the nonlinear activation function layer is used to input a convolution layer with the size of the convolution kernel set to 3 × 3 × 3, which is used to extract the feature information of the target area of interest in the data. The superposition of the two three-dimensional convolution layers deepens the depth of the network. Such a structure can greatly reduce the computational complexity without reducing the network performance in the image segmentation task of cervical cancer. The obtained structural pattern of the cervical cancer lesion feature extraction network is shown in Figure 2, and the final flowchart of the algorithm is shown in Figure 3.

### 2.4. MRI Image Quality Evaluation Based on Artificial Intelligence 3D-CNN Algorithm

The data set used in this study is the BraTS 2017 data set. The experimental environment uses the 12G TITAN X device and uses the deep learning framework TensorFlow and Keras to conduct relevant training under the Ubuntu 16.04.

In this study, the lesion range of cervical cancer lesion (set as Q) delineated by three radiologists was compared with the lesion area (set as W) determined by the image segmentation of the artificial intelligence algorithm. By fitting the coincidence degree between the real cervical cancer lesion area (set as A) and the cervical cancer lesion area (set as C) determined by the artificial intelligence algorithm, the accuracy (Dice), sensitivity, and specificity of the algorithm for the diagnosis of multimodal MRI images of cervical cancer patients were calculated, and the equations were as follows:

(11) $$\begin{array}{c} \text{Dice}=\frac{|A\cap C|}{|A|+|C|/2}, \end{array}$$

(12) $$\begin{array}{c} \text{Sensitivity}=\frac{|A\cap C|}{|A|}, \end{array}$$

(13) $$\begin{array}{c} \text{Specificity}=\frac{|B\cap D|}{|B|}. \end{array}$$

*B* and *D* represent other locations outside the real cervical cancer lesion area and other locations outside the cervical cancer lesion area determined by the artificial intelligence algorithm. In addition, according to the different structural groups of cervical cancer tumors, three tumor regions with overlapping parts are divided, namely, whole-area tumors (WT), core tumor areas (CT), and enhanced tumor areas (ET).

On this basis, this study also introduces Hausdorff (Haus) distance as an auxiliary evaluation index for the advantages and disadvantages of the algorithm [15, 16]. Haus distance can be understood as the maximum of the shortest distance from a point set to another point set. The mathematical expression is shown as follows:

(14) $$\begin{array}{c} Haus=\max\left\{H\left(A,C\right),H\left(C,A\right)\right\}. \end{array}$$

### 2.5. Effect Analysis of MRI Image Diagnosis Based on Artificial Intelligence 3D-CNN Algorithm

In this study, the diagnostic results of multimodal MRI images of cervical cancer and the actual pathological results were compared and analyzed by comparing the traditional CNN algorithm and artificial intelligence 3D-CNN algorithm. The diagnostic accuracy of cervical cancer patients before and after treatment by the two algorithms was calculated, and the application value of the artificial intelligence 3D-CNN algorithm in the diagnosis of multimodal MRI images of cervical cancer was comprehensively evaluated.

### 2.6. Statistical Method

The experimental data were analyzed by SPSS19.0 statistical software. Measurement data were expressed as mean ± standard deviation ($\bar{\text{x}}$ ± s). A *t*-test was used to compare the mean between groups. Count data were expressed as percentage (%). *χ*^2^ test was used to compare the difference between groups. *P* *<* 0.05 was considered statistically significant.

## 3. Results

### 3.1. Multimodal MRI Scan Parameters of Patients

**Figure 4:**
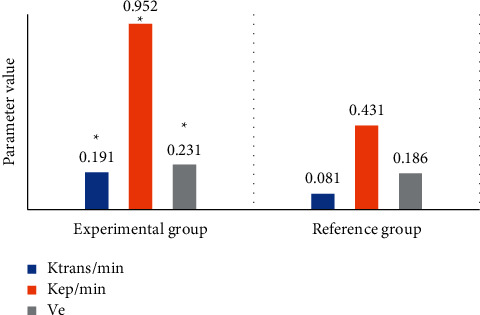
Comparison of DCE-MRI scanning parameters between the two groups of patients. (*Note*.  ^*∗*^ represents a significant difference relative to the CNN algorithm (*P* < 0.05).

**Figure 5:**
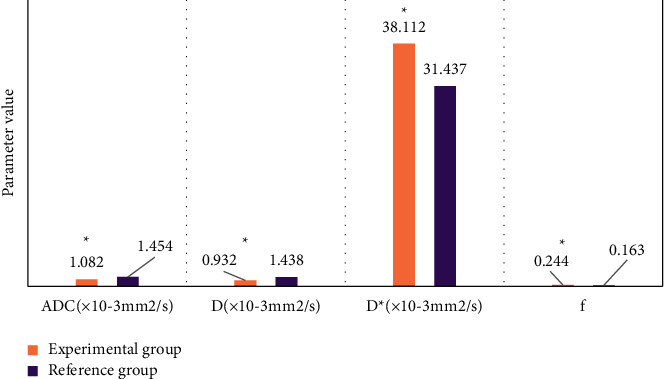
Comparison of IVIM scanning parameters between the two groups of patients. (*Note*.  ^*∗*^ represents a significant difference relative to the CNN algorithm (*P* < 0.05).

Figure 4 shows the comparison of DCE-MRI scanning parameters between the two groups, and Figure 5 shows the comparison of IVIM scanning parameters between the two groups. The Ktrans, Kep, and Ve values of DCE-MRI scanning in the experimental group were 0.191 min, 0.952 min, and 0.231, respectively, while those in the reference group were 0.081 min, 0.431 min, and 0.186, respectively. The apparent diffusion coefficient (ADC), true diffusion coefficient (D), false diffusion coefficient (D*∗*), and perfusion fraction (*f*) of IVIM scanning in the experimental group were 1.082 × 10–3 mm^2^/*s*, 0.932 × 10–3 mm^2^/*s*, 38.112 × 10–3 mm^2^/*s*, and 0.244, respectively, while the corresponding parameter values of the reference group were 1.454 × 10–3 mm^2^/*s*, 1.438 × 10–3 mm^2^/*s*, 31.437 × 10–3 mm^2^/*s*, and 0.163, respectively. The differences between the two parameters were significant (*P* *<* 0.05).

### 3.2. Performance Test Results of Artificial Intelligence 3D-CNN Algorithm

**Figure 6:**
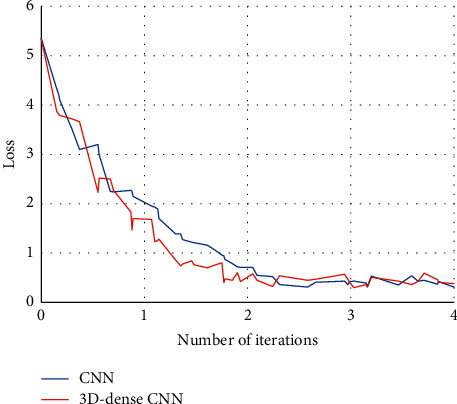
Loss training results analysis of two algorithms.

Figure 6 indicates the loss curves of the two algorithms. The blue curve represents the loss change of the traditional CNN, and the red curve represents the loss change of the artificial intelligence 3D-CNN algorithm. The comparison revealed that the convergence speed of the network was accelerated after the continuous convolution layer was replaced by the dense connection unit.

**Figure 7:**
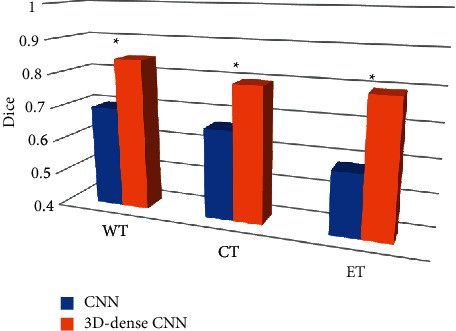
Dice analysis of the two algorithms under the training set. (*Note*.  ^*∗*^ indicates a significant difference compared with the CNN algorithm (*P* < 0.05).

Figure 7 shows the Dice result analysis of the two algorithms under the training set. The results showed that the prediction results of the three regional segmentations of the two networks were compared. The dice average value of the prediction results between 30,000 and 40,000 times was selected as a reference. The artificial intelligence 3D-CNN algorithm significantly improved the accuracy of the segmentation results compared with the traditional CNN (*P* *<* 0.05).

### 3.3. MRI Image Analysis of Cervical Cancer Patients Based on Artificial Intelligence 3D-CNN Algorithm

**Figure 8:**
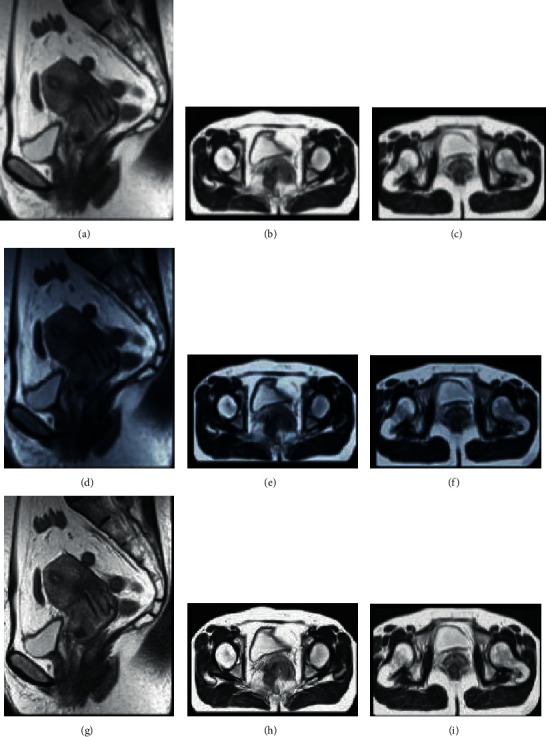
MRI images processed by different algorithms. (a) T2W1 image of normal abdominal MRI sagittal position. (b) T1W1 image of normal abdominal MRI transverse position. (c) T2W1 image of normal abdominal MRI transverse position. (d) T2W1 image of abdominal MRI sagittal position of traditional CNN. (e) T1W1 image of abdominal MRI transverse position of traditional CNN. (f) T2W1 image of abdominal MRI transverse position of traditional CNN. (g) T2W1 image of abdominal MRI sagittal position of artificial intelligence 3D-CNN algorithm. (h) T1W1 image of abdominal MRI transverse position of artificial intelligence 3D-CNN algorithm. (i) T2W1 image of abdominal MRI transverse position of artificial intelligence 3D-CNN algorithm.

Figure 8 shows the MRI images processed by different algorithms, including the sagittal T2W1 image, the transverse T1W1 image, and the T2W1 image. Compared with the multimodal MRI images before the algorithm processing, the clarity and the recognition performance of the MRI images processed by the traditional CNN algorithm and the artificial intelligence 3D-CNN algorithm were significantly improved, so that the structure of the parauterine, adjacent organs, pelvic wall, and lymph nodes on the transverse position was displayed more clearly, and the sagittal image can show the relationship between the lesion and the vagina, bladder, and rectum more intuitively.

**Figure 9:**
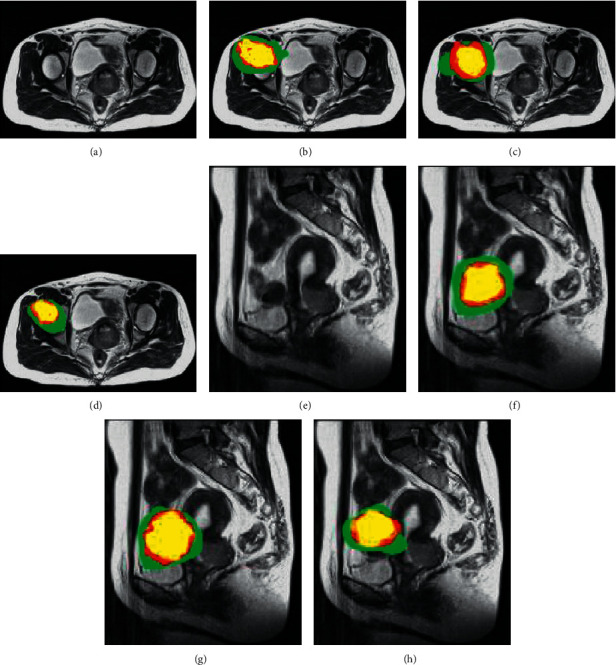
Visual comparison results of multimodal MRI images in the experiment. (*Note*. A and E are the transverse and sagittal images of multimodal MRI in patients under normal conditions. B and F are the prediction results of cervical cancer lesions in transverse and sagittal planes delineated by doctors. C and G are the prediction results of cervical cancer lesions in transverse and sagittal positions of the traditional CNN algorithm. D is the prediction of cervical cancer lesions in transverse and sagittal positions by the artificial intelligence 3D-CNN algorithm).

Figure 9 indicates the visualization results of multimodal MRI images in the experiment, including the images of transverse and sagittal positions. Compared with the multimodal MRI images before the algorithm processing, the visualization results of tumor lesions obtained by the traditional CNN algorithm and the artificial intelligence 3D-CNN algorithm accounted for a similar proportion of the tumor core region (yellow) and the whole region (green). Compared with the true value label, the segmentation results of the three regions by the traditional CNN were fuzzy, and there were many misclassification phenomena in the whole area of the tumor. Most of the right upper corner of the enhanced tumor area (red) was not recognized. After adding the residual network of artificial intelligence 3D-CNN, the misclassification of the whole area of the tumor was significantly reduced, and the segmentation effect of the enhanced tumor area was also improved.

### 3.4. MRI Image Quality Evaluation Based on Artificial Intelligence 3D-CNN Algorithm

**Figure 10:**
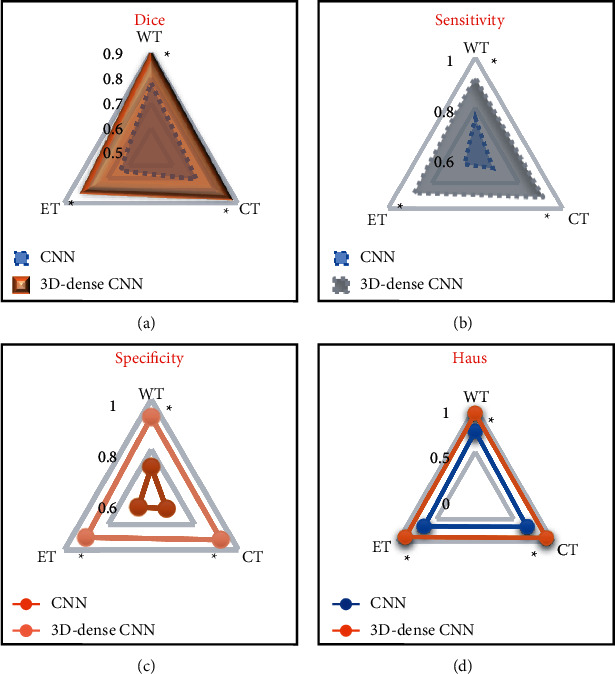
Image processing quality evaluation index comparison chart of different algorithms. (a) Dice value comparison diagram. (b) Sensitivity value comparison chart. (c) Specificity value comparison diagram. (d) Haus distance comparison chart.  ^*∗*^ represents a significant difference relative to the CNN algorithm (*P* < 0.05).

The differences between the diagnostic Dice value, sensitivity, specificity, and Haus distance indexes of multimodal MRI image processing of traditional CNN algorithm and artificial intelligence 3D-CNN algorithm were compared, and the obtained results are shown in Figure 10. The Dice values of WT, ET, and CT regions of the multimodal MRI images processed by the traditional CNN algorithm were 0.78, 0.71, and 0.64, respectively. The sensitivity values were 0.76, 0.68, and 0.65, respectively. The specificity values were 0.89, 0.86, and 0.81, respectively. The Haus distances were 0.73, 0.67, and 0.66, respectively. The Dice values of WT, ET, and CT regions of multimodal MRI images processed by artificial intelligence 3D-CNN algorithm were 0.78, 0.71, and 0.64, respectively. The sensitivity values were 0.92, 0.91, and 0.88, respectively. The specificity values were 0.93, 0.92, and 0.9 l, respectively. The Haus distances were 0.93, 0.92, and 0.90, respectively. The various index data of the two algorithms were significantly different (*P* *<* 0.05).

### 3.5. Evaluation of MRI Image Diagnosis Effect Based on Artificial Intelligence 3D-CNN Algorithm

**Figure 11:**
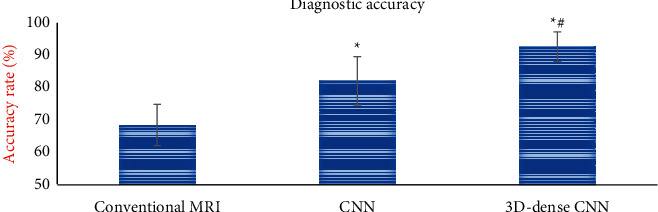
Comparison of image diagnostic accuracy of different algorithms. (*Note*.  ^*∗*^ represents a significant difference in diagnostic accuracy compared with conventional multimodal MRI images (*P* *<* 0.05). # shows a significant difference compared with the CNN algorithm (*P* *<* 0.05).

From Figure 11, the diagnostic accuracy of the conventional multimodal MRI image diagnosis, the multimodal MRI image diagnosis processed by the traditional CNN algorithm, and the multimodal MRI image diagnosis processed by the artificial intelligence 3D-CNN algorithm were 68.65 ± 6.44%, 82.45 ± 7.54%, and 93.11 ± 4.65%, respectively. Compared with the diagnostic accuracy of the conventional multimodal MRI image, the diagnostic accuracy of the images processed by the two algorithms was significantly increased (*P* *<* 0.05), and the accuracy of the artificial intelligence 3D-CNN algorithm was also significantly higher than that of the traditional CNN algorithm (*P* *<* 0.05).

## 4. Discussion

In recent years, clinical computer-aided diagnosis and treatment methods for various common clinical diseases emerged one after another [17], and deep learning technology gradually replaced supervised learning as an effective computer-aided artificial intelligence medical image segmentation scheme relying on its strong model learning ability and highly automatic extraction of target feature information [18], which was widely used in medical image segmentation fields of brain tumors, lung cancer, liver cancer, breast cancer, and gastric cancer, such as ultrasound, CT, and MRI images [19, 20].

In this study, an artificial intelligence 3D-CNN algorithm was designed for the lesion image characteristics of cervical cancer patients under multimodal MRI and applied to the multimodal MRI image diagnosis of clinical cervical cancer. The results showed that compared with the traditional CNN algorithm, the convergence rate of the loss curve of the artificial intelligence 3D-CNN algorithm was accelerated, and the segmentation accuracy of WT, CT, and ET was significantly improved. In addition, the clarity of multimodal MRI images and the recognition performance of lesions were significantly improved. Under the artificial intelligence 3D-CNN algorithm, the Dice values of WT, ET, and CT regions were 0.78, 0.71, and 0.64, respectively. The sensitivity values were 0.92, 0.91, and 0.88, respectively. The specificity values were 0.93, 0.92, and 0.9 l, respectively. The Haus distances were 0.93, 0.92, and 0.90, respectively. The data of various indicators were significantly better than those of the traditional CNN algorithm (*P* *<* 0.05). In addition, the diagnostic accuracy of the artificial intelligence 3D-CNN algorithm was 93.11 ± 4.65%, which was also significantly higher than that of the traditional CNN algorithm (82.45 ± 7.54%) (*P* *<* 0.05). This is consistent with the research results of Chen et al. [21]. The diagnostic accuracy of the 3D-CNN algorithm is better than that of the traditional CNN algorithm. But the relevant optimization procedures of the artificial intelligence 3D-CNN algorithm are very complex and need further in-depth study.

## 5. Conclusions

In this study, an artificial intelligence 3D-CNN algorithm is designed for the imaging characteristics of cervical cancer patients under multimodal MRI, and it is applied to the multimodal MRI imaging diagnosis of clinical cervical cancer. The results showed that the recognition and segmentation ability of multimodal MRI images based on artificial intelligence 3D-CNN algorithm for cervical cancer lesions was significantly improved, which could significantly enhance the clinical diagnosis rate of cervical cancer. However, there are still some shortcomings in this study. When discussing the fusion of multimodal MRI images, it was not found that a 3D-CNN image fusion scheme highlighted the characteristics of each modal MRI image. In addition, the algorithm in this study is not very good for the whole tumor segmentation edge, and it needs to be further optimized in the future. In conclusion, multimodal MRI images based on artificial intelligence 3D-CNN algorithm can significantly improve the clinical diagnostic accuracy of cervical cancer, which brings certain reference value for improving the clinical diagnosis and treatment efficiency of cervical cancer patients.

## Data Availability

The data used to support the findings of this study are available from the corresponding author upon request.

## References

[1] Pedersen K., Fogelberg S., Thamsborg L. H. (2018). An overview of cervical cancer epidemiology and prevention in Scandinavia. *Acta Obstetricia et Gynecologica Scandinavica*.

[2] Moga M., Dimienescu O., Arvatescu C., Mironescu A., Dracea L., Ples L. (2016). The role of natural polyphenols in the prevention and treatment of cervical cancer-an overview. *Molecules*.

[3] De Strooper L. M. A., Berkhof J., Steenbergen R. D. M. (2018). Cervical cancer risk in HPV‐positive women after a negative FAM19A4/mir124‐2 methylation test: a post hoc analysis in the POBASCAM trial with 14 year follow‐up. *International Journal of Cancer*.

[4] Tamura K., Hasegawa K., Katsumata N. (2019). Efficacy and safety of nivolumab in Japanese patients with uterine cervical cancer, uterine corpus cancer, or soft tissue sarcoma: multicenter, open‐label phase 2 trial. *Cancer Science*.

[5] Hong D. S., Concin N., Vergote I. (2020). Tisotumab vedotin in previously treated recurrent or metastatic cervical cancer. *Clinical Cancer Research*.

[6] Santin A. D., Deng W., Frumovitz M. (2020). Phase II evaluation of nivolumab in the treatment of persistent or recurrent cervical cancer (NCT02257528/NRG-GY002). *Gynecologic Oncology*.

[7] Lakhman Y., Akin O., Park K. J. (2013). Stage IB1 cervical cancer: role of preoperative MR imaging in selection of patients for fertility-sparing radical trachelectomy. *Radiology*.

[8] Minnaar C. A., Kotzen J. A., Ayeni O. A. (2019). The effect of modulated electro-hyperthermia on local disease control in HIV-positive and -negative cervical cancer women in South Africa: early results from a phase III randomised controlled trial. *PLoS One*.

[9] Kalaghchi B., Abdi R., Amouzegar-Hashemi F., Esmati E., Alikhasi A. (2016). Concurrent chemoradiation with weekly paclitaxel and cisplatin for locally advanced cervical cancer. *Asian Pacific Journal of Cancer Prevention*.

[10] Murakami N., Kato S., Nakano T. (2016). A phase I/II clinical trial for the hybrid of intracavitary and interstitial brachytherapy for locally advanced cervical cancer. *BMC Cancer*.

[11] Knott K. D., Seraphim A., Augusto J. B. (2020). The prognostic significance of quantitative myocardial perfusion: an artificial intelligence based approach using perfusion mapping. *Circulation*.

[12] Gates E. D. H., Lin J. S., Weinberg J. S. (2020). Imaging-based algorithm for the local grading of glioma. *American Journal of Neuroradiology*.

[13] Albizu A., Fang R., Indahlastari A. (2020). Machine learning and individual variability in electric field characteristics predict tDCS treatment response. *Brain Stimulation*.

[14] Papp L., Spielvogel C. P., Grubmüller B. (2021). Supervised machine learning enables non-invasive lesion characterization in primary prostate cancer with [68Ga]Ga-PSMA-11 PET/MRI. *European Journal of Nuclear Medicine and Molecular Imaging*.

[15] Pereira S., Pinto A., Alves V., Silva C. A. (2016). Brain tumor segmentation using convolutional neural networks in MRI images. *IEEE Transactions on Medical Imaging*.

[16] Su J. H., Thomas F. T., Kasoff W. S. (2019). Thalamus Optimized Multi Atlas Segmentation (THOMAS): fast, fully automated segmentation of thalamic nuclei from structural MRI. *NeuroImage*.

[17] Aghili M., Andalib B., Karimi Moghaddam Z., Maddah Safaie A., Amoozgar Hashemi F., Mousavi Darzikolaie N. (2018). Concurrent chemo- radiobrachytherapy with cisplatin and medium dose rate intra- cavitary brachytherapy for locally advanced uterine cervical cancer. *Asian Pacific Journal of Cancer Prevention: Asian Pacific Journal of Cancer Prevention*.

[18] Banks T. I., von Eyben R., Hristov D., Kidd E. A. (2018). Pilot study of combined FDG-PET and dynamic contrast-enhanced CT of locally advanced cervical carcinoma before and during concurrent chemoradiotherapy suggests association between changes in tumor blood volume and treatment response. *Cancer Medicine*.

[19] Benard V. B., Saraiya M., Greek A. (2014). Overview of the CDC Cervical Cancer (Cx3) Study: an educational intervention of HPV testing for cervical cancer screening. *Journal of Women’s Health*.

[20] Downey K., Jafar M., Attygalle A. D. (2013 6). Influencing surgical management in patients with carcinoma of the cervix using a T2- and ZOOM-diffusion-weighted endovaginal MRI technique. *British Journal of Cancer*.

[21] Chen C., Bai W., Davies R. H. (2020). Improving the generalizability of convolutional neural network-based segmentation on CMR images. *Frontiers in Cardiovascular Medicine*.

